# Leveraging Macrophage Metabolic Reprogramming for Enhanced Anti‐Tumor Immunity

**DOI:** 10.1002/advs.202520903

**Published:** 2026-04-10

**Authors:** Zhiyun Liu, Lingyao Zeng, Xulin Gan, Yilin Zhou, Han Shen, Dan Wu, Xin Shou, Minmin Jiang, Liyun Shi

**Affiliations:** ^1^ Zhejiang Chinese Medical University Hangzhou China; ^2^ Key Laboratory of Artificial Organs and Computational Medicine in Zhejiang Province Institute of Translational Medicine Zhejiang Shuren University Hangzhou China; ^3^ Yuhang Institute of Medical Science Innovation and Transformation, Zhejiang Hangzhou China

**Keywords:** macrophage polarization, metabolic reprogramming, targeted drug delivery, tumor microenvironment, tumor‐associated macrophages (TAMs)

## Abstract

Tumor‐associated macrophages (TAMs) are central regulators of the tumor microenvironment (TME), with their metabolic states critically influencing tumor progression or regression. Although reprogramming TAM metabolism is a promising therapeutic avenue, clinical translation remains challenging due to the oversimplified understanding of macrophage plasticity. To bridge these gaps, we first provide an in‐depth analysis of the metabolic signatures and functional heterogeneity of TAMs, highlighting key pathways—glycolysis, fatty acid oxidation, and amino acid metabolism—that govern TAM functional diversity. Building on this foundation, we offer a comprehensive overview of current therapeutic strategies targeting critical metabolic regulatory nodes in TAMs and explore future directions for their clinical translation. Ultimately, we propose that precisely modulating the metabolic networks of TAMs can effectively reprogram their immunosuppressive functions, thereby opening new avenues for advancing cancer immunotherapy.

## Graphical abstract

Tumor‐associated macrophages (TAMs) are key regulators of the tumor microenvironment (TME), with their metabolic states playing a critical role in tumor progression or regression. This review summarizes current understanding of TAM metabolic plasticity alongside cutting‐edge bioengineering innovations, outlining a roadmap for transforming the immunosuppressive TME into a pro‐inflammatory environment, thereby paving the way for the next generation of macrophage‐centered immunotherapies.

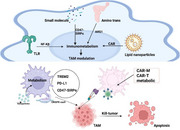

## 1 Introduction

**FIGURE 1:**
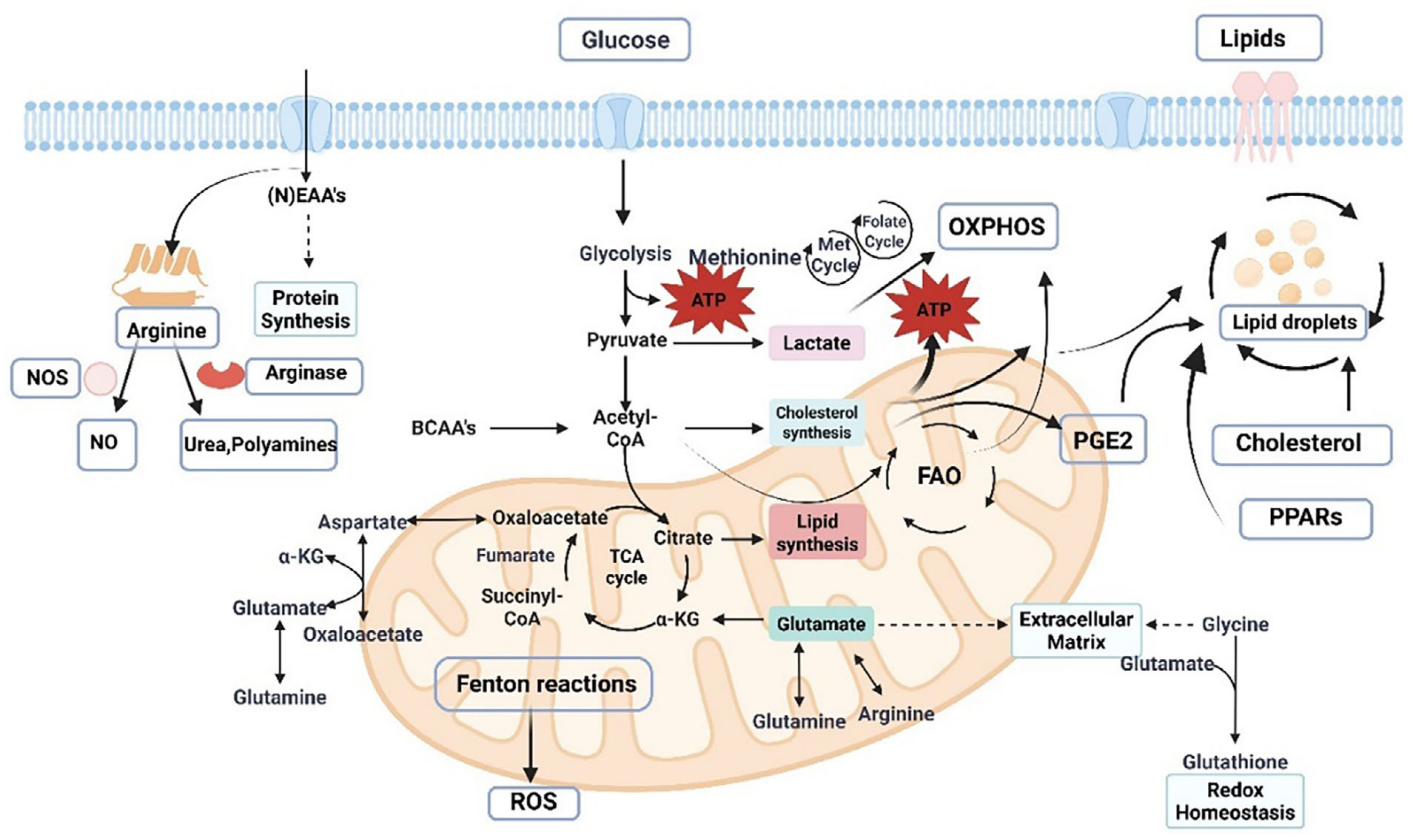
Major metabolic pathways in macrophages. Created using Biorender (https://biorender.com/).

Immunometabolism is a rapidly evolving field that investigates how immune cells metabolize nutrients to fuel their growth and function, with metabolic reprogramming exerting broad impacts on cellular functions and disease progression [1, 2]. Macrophages, phagocytic cells derived from monocytes and renowned for their plasticity and functional versatility, play a crucial role in clearing aged or apoptotic cells, engulfing immune complexes and pathogens, and maintaining innate immune homeostasis. Depending on the stimuli present in the tumor microenvironment (TME), macrophages can polarize into either an immunosuppressive M2‐like phenotype or a pro‐inflammatory M1‐like phenotype, demonstrating considerable functional plasticity [3]. Critically, this polarization is governed by distinct metabolic programs (Figure 1). M1‐like (anti‐tumor) macrophages primarily rely on aerobic glycolysis to generate immunomodulatory metabolites, whereas M2‐like (pro‐tumor) macrophages preferentially utilize oxidative phosphorylation and fatty acid oxidation [4]. These metabolic reprogramming is not merely a consequence but a central regulator of macrophage phenotype, establishing and maintaining their polarization states, thereby profoundly influencing tumor progression and anti‐tumor immune responses.

The tight metabolic regulation of macrophage plasticity presents a promising therapeutic opportunity to enhance anti‐tumor immunity. Strategies targeting these critical metabolic nodes encompass a broad spectrum of approaches [5, 6, 7, 8, 9]. Small‐molecule inhibitors of key metabolic enzymes can directly rewire intracellular metabolic flux and shift macrophage polarization, whereas immunometabolism modulators reprogram the metabolic landscape of the tumor microenvironment. Notably, recent advances in CRISPR‐Cas9 technology have enabled precise genetic editing of metabolic checkpoints, such as disrupting *Pik3cg* (PI3K𝛾) to drive M2‐to‐M1 repolarization and destroy immunosuppressive niche [10]. Encouragingly, preclinical and early clinical translations of these metabolic reprogramming strategies have yielded promising results, particularly when combined with established immunotherapies like immune checkpoint blockade [11, 12, 13, 14]. However, translating these insights into durable clinical responses faces several critical hurdles. These include the profound metabolic heterogeneity of tumor‐associated macrophages (TAMs), the complex metabolite‐driven crosstalk between tumor cells and macrophages that fuels immune suppression, and the ongoing challenge of achieving efficient, targeted delivery of metabolic inhibitors to specific macrophage subsets in vivo. Addressing these issues is essential for unlocking the full potential of macrophage‐based immunometabolic therapies.

In this review, we systematically describe the metabolic reprogramming of TAMs, detailing how key metabolic pathways fundamentally dictate their phenotypes and functions. We explore the remarkable heterogeneity of TAMs revealed by single‐cell and spatial multi‐omics technologies, highlighting that the functional plasticity of distinct TAM subsets is governed by epigenetic modifications and signals from the tumor microenvironment. Building on this mechanistic foundation, we critically assess emerging therapeutic strategies that target these metabolic nodes, ranging from small‐molecule modulators to advanced platforms such as nanotechnology, CRISPR‐Cas9 gene editing, and CAR‐macrophages. Inspired by these insights, we propose that precisely reprogramming tumor‐associated macrophages and transforming them into potent effectors could serve as a next‐generation immunotherapy approach against cancer.

## 2 Macrophage Polarization and Function Diversity

Macrophages exhibit remarkable functional plasticity, governed by dynamic epigenomic and metabolic reprogramming in response to microenvironmental stimuli. A hallmark of tumorigenesis is the substantial infiltration of these macrophages, termed TAMs, into tumor tissue. TAMs are pivotal component of the TME, existing primarily as a dysfunctional, pro‐tumor population that orchestrates immunosuppressive effects by modulating immune cells, stromal components, and the tumor vasculature [15, 16, 17].

**FIGURE 2:**
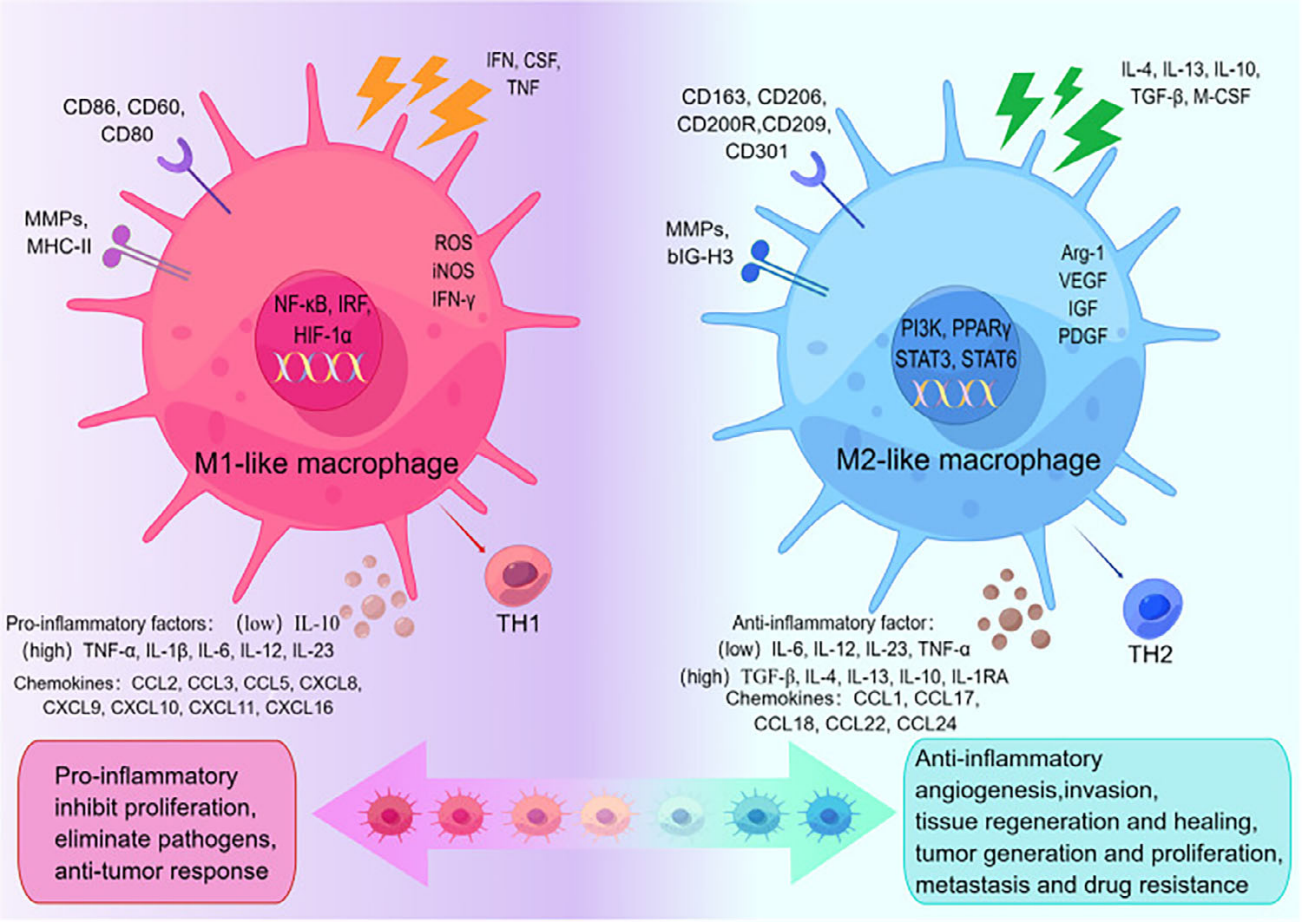
Macrophage M1/M2 polarization. M1‐like and M2‐like macrophages are characterized by distinct cell surface markers and secretory profiles. Their functional and phenotypic divergences underpin their contrasting roles within the tumor microenvironment. Reproduced with permission [22]. Copyright 2024, Springer Nature.

The spectrum of macrophages is often divided into two opposing phenotypes (Figure 2) [18]. The M1‐like phenotype is typically induced by microbial products, such as lipopolysaccharide (LPS), and pro‐inflammatory cytokines like interferon‐gamma (IFN‐γ). These macrophages are potent anti‐tumor effectors, characterized by the secretion of pro‐inflammatory cytokines (e.g., IL‐12 and TNF‐α) and high‐level production of nitric oxide (NO) and reactive oxygen species (ROS) [19, 20, 21]. Their secretome is rich in chemokines (e.g., CXCL9, CXCL10, and CXCL11) that are critical for recruiting and activating T helper 1 (Th1) cells, thereby amplifying a cytotoxic immune response that suppresses tumor growth [22, 23, 24].

In contrast, the M2‐like phenotype is driven by Th2‐associated cytokines, notably interleukin‐4 (IL‐4) and interleukin‐13 (IL‐13). These macrophages are critically involved in mediating immunosuppressive responses and promoting tissue remodeling processes. Their functional profile encompasses the secretion of anti‐inflammatory cytokines, including interleukin‐10 (IL‐10), elevated expression of scavenger receptors such as CD163 and the mannose receptor CD206, as well as pronounced arginase‐1 (Arg‐1) enzymatic activity. By inhibiting Th1‐mediated immune response, M2‐like macrophages directly facilitate tumor cell proliferation, angiogenesis, and resistance to therapeutic interventions [25, 26]. The M2‐like macrophage population can be further subdivided into distinct subtypes (e.g., M2a, M2b, and M2c) based on their specific responses to different stimuli. Notably, M2a macrophages are induced by interleukins IL‐4 and IL‐13, M2b macrophages are activated by IL‐1β, and M2c macrophages are stimulated by anti‐inflammatory agents including glucocorticoids, IL‐10, and transforming growth factor‐beta (TGF‐β). Notably, all three subtypes exhibit elevated expression of CD11 and secrete anti‐inflammatory cytokines such as IL‐10, as well as chemokines including CCL24 and CCL22, which contribute to the suppression of pro‐inflammatory immune responses [27]. Thus, the functional polarization of macrophages, underpinned by distinct molecular signatures, critically shapes their interactions with other cellular components within the TME.

## 3 Metabolic Characterization of TAMs

Tumor microenvironment is a metabolically intricate landscape, characterized by hypoxia, nutrient deprivation, and an accumulation of metabolic byproducts like lactate, largely driven by the metabolic property of cancer cells [28]. Immune cells infiltrating the TME must adapt to this challenging environment, and their own metabolic reprogramming is a primary determinant of their function.

### 3.1 Glucose Metabolism in Macrophage Polarization

#### 3.1.1 Glycolytic and Oxidative Phosphorylation Signatures in M1 Macrophages

Upon stimulation with signals like LPS and IFN‐γ, M1‐like macrophages undergo a profound metabolic alteration that dramatically upregulate glycolysis [29]. This glycolytic switch is not merely for energy production; it is a strategic rewiring to support a robust pro‐inflammatory response [30, 31]. Concurrently, nitric oxide (NO) produced by iNOS further suppresses mitochondrial oxidative phosphorylation (OXPHOS) by nitrosylating electron transport chain components, while glycolytic flux is diverted into the pentose phosphate pathway to generate NADPH for ROS production via NADPH oxidase [31, 32].

**FIGURE 3:**
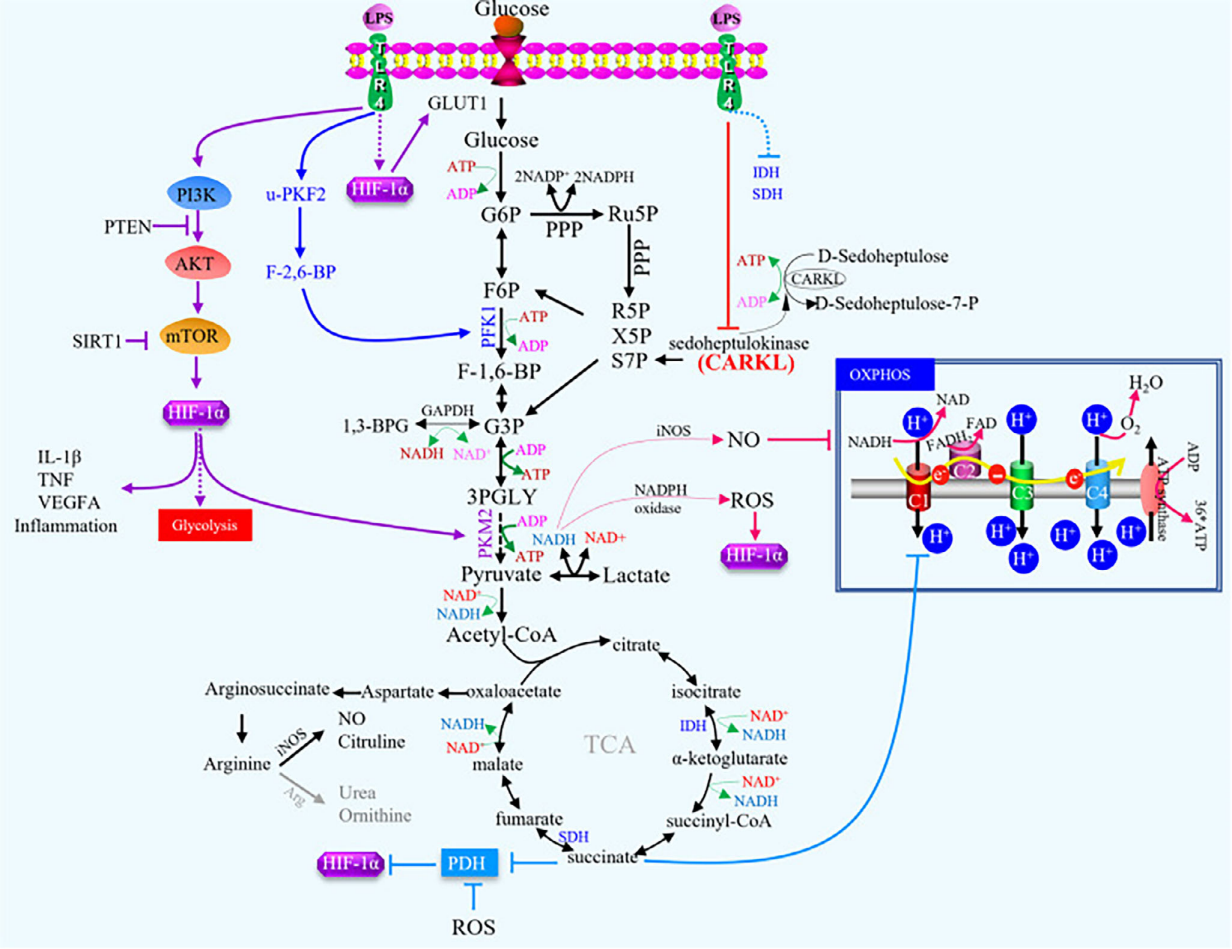
Complex crosstalk among glycolysis, TCA cycle, and OXPHOS contributes to the glucose metabolic signature of macrophages and regulates their polarization. Reproduced with permission [17]. Copyright 2023, Springer Nature.

This metabolic rewiring is functionally coupled to two critical breaks in the TCA cycle, which result in the accumulation of specific immunomodulatory metabolites [33] (Figure 3). The first break occurs after citrate, which is exported to the cytosol to serve as a precursor for fatty acid synthesis‐essential for membrane expansion during phagocytosis‐and the production of inflammatory mediators. The second break occurs at succinate dehydrogenase (SDH), leading to the accumulation of succinate. Succinate acts as a potent pro‐inflammatory signal by stabilizing HIF‐1α, thereby creating a positive feedback loop that reinforces the glycolytic phenotype and enhances the production of IL‐1β [30, 34]. Metabolomic profiling confirms this signature: early‑phase macrophages accumulate glycolytic intermediates (glucose‑6‑phosphate, fructose‑1,6‑bisphosphate), increase lactate production, and elevate pentose phosphate pathway (PPP) intermediates (ribose‑5‑phosphate, NADPH/NADP^+^ ratio), collectively providing energy and biosynthetic precursors for effector functions [35, 36]. This metabolic architecture directly fuels the anti‑tumor activities of M1‑like macrophages. These cells exhibit potent cytotoxicity, phagocytose tumor cells, enhance antigen presentation, and secrete pro‑inflammatory cytokines (IL‑6, IL‑12, TNF) and chemokines that recruit and activate CD8^+^ T cells and natural killer (NK) cells, orchestrating an anti‑tumor attack and shaping an immunologically “hot” TME [37].

#### 3.1.2 Glycolytic and Oxidative Phosphorylation Signatures in M2 Macrophages

M2‐like macrophages, polarized by cytokines such as IL‐4 and IL‐13, rely on an intact TCA cycle and robust OXPHOS as their primary mode of energy production [33]. This metabolic program supports sustained, long‐term functions like tissue repair, angiogenesis, and immunosuppression. M2 macrophages fuel oxidative metabolism primarily through fatty acid oxidation (FAO) and glutamine [30]. The enzyme PFKFB1, which is highly expressed in M2 macrophages, limits glycolytic flux, thereby favoring OXPHOS [31, 38]. This metabolic pattern, often driven by the activation of AMPK, supports their pro‐tumorigenic role. Consequently, M2‐like TAMs secrete anti‐inflammatory cytokines like IL‐10 and a repertoire of growth factors (e.g., PDGF, TGF‐β) that directly promote tumor cell proliferation and diminish adaptive immune responses [16, 22, 39, 40]. Enriched in hypoxic and perivascular regions of the TME, M2‐like TAMs drive angiogenesis by secreting factors such as VEGF and MMPs [37]. This metabolic behavior provides the bioenergetic foundation for their immunosuppressive and tissue‐remodeling functions, which collectively create a TME conducive to tumor growth and metastasis.

Hexokinase‐2 (HK2), the rate‐limiting enzyme of glycolysis, has emerged as more than a simple metabolic gatekeeper. Recent studies have revealed its dual role as a sophisticated signaling hub that integrates metabolic cues with macrophage function. First, in the context of inflammation, HK2 plays a key role in lactate‐derived histone lactylation (H3K18la) [41]. This epigenetic modification enhances the expression of glycolytic genes, including HK2 itself, thereby locking in the M1 pro‐inflammatory phenotype. Second, HK2 can act as a metabolic sensor. In colorectal cancer models, TAMs utilize HK2 to sense fructose, which triggers an unexpected protein–protein interaction that suppresses calcium signaling and attenuates NLRP3 inflammasome activation, thereby ultimately impeding M1 polarization and fostering an immunosuppressive TME [42]. This dual functionality positions HK2 as a critical coordinator of macrophage polarization and a rational therapeutic target for reprogramming TAMs using agents like the glucose analog 2‐deoxy‐D‐glucose (2‐DG) [43].

### 3.2 Fatty Acid Oxidation

**FIGURE 4:**
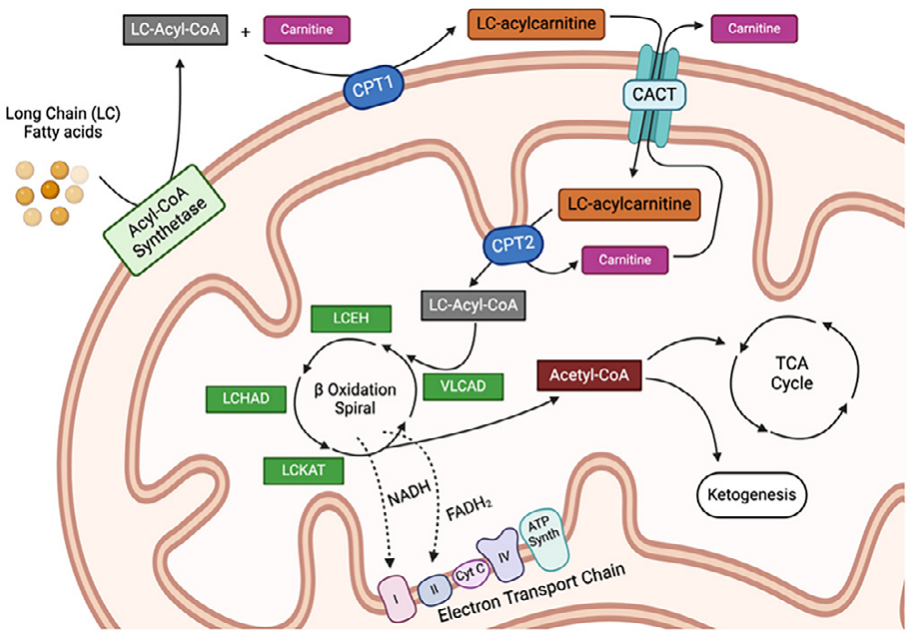
Intracellular transport and oxidation metabolism of long chain fatty acids. Reproduced with permission [46]. Copyright 2024, Frontiers Media S.A.

The metabolic signature of M2‐like macrophages is also dominated by the uptake and catabolism of exogenous lipids through FAO. This reliance on FAO is not merely for energy production but also serves as a powerful driver of their immunosuppressive and tissue‐remodeling functions. The FAO dependent phenotype of macrophages is supported by a sophisticated molecular machinery. The process begins with the enhanced uptake of lipids from the TME, mediated by scavenger receptors such as CD36. High expression of CD36 on TAMs facilitates the accumulation of intracellular lipid droplets‐a hallmark of their pro‐tumorigenic state, which promotes the secretion of immunosuppressive factors like IL‐10 [44, 45]. Once inside the cell, long‐chain fatty acids are transported into the mitochondria via the rate‐limiting carnitine shuttle system, a process dependent on the enzymes CPT1a and CPT2 (Figure 4). Within the mitochondria, they undergo β‐oxidation to generate a steady supply of acetyl‐CoA, NADH, and FADH_2_ [46]. This output fuels the TCA cycle and OXPHOS, providing the sustained energy required for M2‐like functions such as anti‐inflammatory cytokine secretion and tissue repairing.

These lipid metabolic programs are under tight transcriptional control. The master regulator of the M2‐like lipid metabolic state is the nuclear receptor PPAR‐γ, often acting in concert with STAT6 signaling to drive the expression of FAO related genes [47, 48, 49, 50, 51]. Other key transcription factors further orchestrate broader lipid homeostasis. These include Sterol Regulatory Element‐Binding Protein (SREBP1), which governs lipid synthesis [52, 53], and Liver X Receptors (LXRs), which control cholesterol efflux [54]. The dysregulation of these transcription factors in TAMs is critical for establishing and maintaining the tumor‐supportive microenvironment.

Beyond bioenergetics, FAO plays a key functional role by fueling the TCA cycle to generate acetyl‐CoA, the primary substrate for histone acetylation, thereby directly linking metabolism to epigenetic regulation [55]. This provides a direct link between metabolism and the epigenome, whereby increased FAO reinforces a transcriptional program that promotes an M2‑like, tolerogenic state characterized by poor antigen‐presenting capacity [46]. Furthermore, tumor cells actively exploit this metabolic dependency. For example, ovarian cancer cells can promote cholesterol efflux from macrophages, which exhausts lipid rafts and enhances IL‐4‐mediated M2 reprogramming while suppressing anti‐tumor IFN‐γ signaling [56]. This metabolic crosstalk drives functional specialization of lipid‑fueled TAMs, enabling them to execute distinct pro‐tumor functions based on their location within the TME‐promoting invasion, metastasis, and angiogenesis [57].

However, the deep reliance on lipid metabolism represents a key therapeutic vulnerability in TAMs. Targeting key nodes in this network, such as inhibiting lipid uptake (anti‐CD36 strategies), blocking FAO (e.g., CPT1a inhibitor etomoxir), or modulating critical regulators (e.g., PPAR‐γ inhibitors, SREBP1 inhibitors, LXR agonists), offers a rational and powerful approach to reprogram TAMs, reverse their immunosuppressive functions, and restore potent anti‐tumor immunity.

### 3.3 Amino Acid Metabolism: Arginine and Glutamine

#### 3.3.1 Arginine Metabolism

Amino acids serve not only as building blocks for protein synthesis but also as dynamic signaling molecules that regulate macrophage metabolism. Among various amino acid pathways, L‐arginine metabolism represents a critical metabolic bifurcation that fundamentally dictates macrophage function [58]. In pro‐inflammatory M1‐like macrophages, the enzyme inducible nitric oxide synthase (iNOS) converts L‑arginine into NO, a potent anti‐tumor and antimicrobial effector molecule. In contrast, M2‐like macrophages highly express Arg‐1, which hydrolyzes L‐arginine into ornithine and urea, thereby fueling pathways that support cell proliferation and tissue remodeling [59].

Within the tumor microenvironment, the metabolic balance is overwhelmingly skewed toward the Arg‐1 driven pathway in tumor‐associated macrophages, establishing Arg‐1 as a critical immunosuppressive checkpoint. The high enzymatic activity of Arg‐1 in TAMs has two profound pro‐tumorigenic consequences. First, it severely exhausts local L‐arginine, starving infiltrating T cells by lacking this essential amino acid [60]. This nutrient deprivation directly impairs T cell proliferation, cytokine production, T‐cell receptor (TCR) signaling, and induces T cell anergy, thereby weakening the anti‐tumor immune response [61]. Second, the products of this pathway, such as polyamines derived from ornithine, can directly support tumor cell growth.

Cancer cells themselves can serve as a major source of arginine within the tumor microenvironment. Metabolomic profiling of macrophages, combined with clinical sample analysis, has confirmed that breast cancer cells highly express argininosuccinate synthase 1 (ASS1), a key enzyme involved in arginine synthesis [62]. The arginine secreted by cancer cells is taken up by TAMs, where it fuels polyamine synthesis‐particularly spermine‐and promotes their polarization toward an M2‑like, pro‑tumor phenotype. This polarization subsequently suppresses CD8^+^ T cell function and facilitates tumor immune evasion. Mechanistically, these polyamines drive macrophage polarization and subsequent T cell suppression partly through epigenetic regulation. Arginine metabolism can influence pathways such as p53 mediated signaling, which may induce DNA demethylation at specific loci, including the *PPARG* gene promoter. This epigenetic remodeling upregulates PPAR‑γ expression, thereby reinforcing macrophage polarization toward the M2‑like state [63].

**FIGURE 5:**
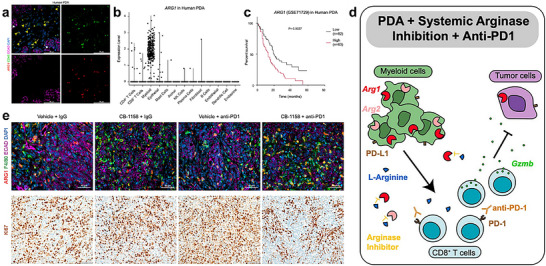
Arginase inhibition in combination with anti‐PD1 immune checkpoint reduces tumor growth. Reproduced with permission [64]. Copyright 2023, eLife Science.

Collectively, systematic macrophage metabolomic studies highlight the central role of the arginine‑polyamine metabolic axis in driving tumor immune evasion. These findings not only elucidate how metabolic reprogramming governs immune cell function through epigenetic mechanisms but also identify novel targets and strategies for metabolism‑based cancer immunotherapy. The central role of Arg‐1 in orchestrating the immunosuppressive niche makes it a primary therapeutic target. Strategies have been validated in preclinical models, where pharmacological inhibition of arginase with small molecules such as CB‐1158 has demonstrated significant efficacy (Figure 5). Notably, combining CB‐1158 with anti‐PD‐1 checkpoint blockade resulted in a significant reduction in tumor growth in pancreatic cancer models [64]. Metabolomic analysis revealed the restoration of arginine levels within the TME of CB‐1158‐treated mice [64]. These findings confirm the Arg‐1 pathway as a key therapeutic vulnerability, offering a powerful strategy to reprogram TAMs and disrupt the immune‐metabolic suppressive microenvironment in cancer.

#### 3.3.2 Glutamine Metabolism

Glutamine, the most abundant amino acid in plasma, serves not only as a key source of carbon and nitrogen for biosynthesis but also as a direct regulator of macrophage polarization. Its metabolism is especially critical for M2‐like macrophage polarization within the tumor microenvironment [65]. These cells depend on glutaminolysis, a process in which glutaminase (GLS) converts glutamine to glutamate, which is then transformed into α‑ketoglutarate (α‑KG) to replenish the TCA cycle. This metabolic flux supports the bioenergetic and biosynthetic demands required for the immunosuppressive and tissue‐remodeling functions of M2‐like macrophages. Consequently, glutamine deprivation inhibits M2 activation [66], while high expression of glutamine synthase (GS), the enzyme responsible for glutamine production, is associated with a pro‐tumorigenic M2 phenotype [67].Therefore, glutamine metabolism plays a central role in fueling the TCA cycle of M2‐like macrophages and maintaining their immunosuppressive function.

In contrast, the glutamine metabolic landscape of M1‑like macrophages is more nuanced. Although glutamine supports their anabolic needs, their pro‑inflammatory and anti‑tumor functions are critically influenced by the intracellular metabolite balance, particularly a lower α‐KG‐to succinate ratio, which favors an anti‑tumor state [68]. This profound dependency on glutamine metabolism across macrophage phenotypes presents an attractive therapeutic vulnerability. The rate‑limiting enzyme in glutaminolysis, GLS, which exists as two primary isoforms, GLS1 and GLS2‐represents a key intervention point. Pharmacological GLS1 inhibitors, such as telaglenastat (CB‐839) have been developed to target this pathway [69]. By blocking GLS activity, these agents can disrupt the metabolism of both tumor cells and M2‐like TAMs, thereby reversing immunosuppression and enhancing the secretion of pro‐inflammatory cytokines.

However, targeting glutamine metabolism presents considerable complexity. Tumor cells and immune cells, including TAMs and T cells, compete for the limited glutamine pool within the TME. Although GLS inhibition can effectively reprogram TAMs, it can also negatively impact the clonal expansion and activation of anti‐tumor CD8^+^ T cells, which also require glutamine for their cytotoxic activities [15]. Therefore, therapeutic strategies must be designed to selectively reprogram immunosuppressive myeloid cells while preserving the activity of essential anti‐tumor lymphocytes. Achieving this requires a precise understanding of the differential glutamine dependence among different cell populations within the TME, which is critical for developing effective metabolic immunotherapies.

### 3.4 Mitochondrial Dynamic and Quality Control

Mitochondria function as dynamic metabolic centers that regulate macrophage polarization by coordinating modifications in their morphology, metabolic signaling pathways, functional outputs, and quality control mechanisms. Such adaptations enable macrophages to modulate their bioenergetic and signaling profiles in response to distinct immunological stimuli. A key feature of this polarization is the dynamic remodeling of the mitochondrial network. In pro‐inflammatory M1‐like macrophages, mitochondria undergo fission, resulting in smaller and fragmented organelles. This structural alteration decreases the efficiency of the electron transport chain (ETC), reduces reliance on OXPHOS, and facilitates a metabolic transition toward aerobic glycolysis [70]. Conversely, anti‐inflammatory M2‐like macrophages predominantly exhibit mitochondrial fusion, which produces an elongated and interconnected network. This fused configuration enhances the efficiency of ETC supercomplexes, thereby supporting robust OXPHOS and FAO to meet the sustained energetic requirements associated with tissue repair and immunoregulatory activities [71].

These structural alterations directly influence metabolic output. In M1 macrophages, the presence of smaller, fragmented mitochondria acts as a significant source of mitochondrial reactive oxygen species (mtROS) [72]. Rather than serving solely as metabolic byproducts, mtROS function as critical signaling molecules that stabilize HIF‐1α and promote the transcription of pro‐inflammatory genes such as *IL‐1β*. This process is fueled by the accumulation of TCA cycle intermediates, notably succinate, which is oxidized by SDH to elevate the mitochondrial membrane potential and amplify mtROS production [73]. In contrast, the fused mitochondria networks observed in M2 macrophages are optimized for efficient ATP production via OXPHOS with minimal ROS leakage, thereby supporting their long‐term functions.

Within the immune response landscape, itaconate has emerged as a pivotal immunometabolite. Specifically, macrophages activated under inflammatory conditions exhibit a marked upregulation of itaconate synthesis, which subsequently exerts complex regulatory effects via multiple molecular pathways [74]. Central to this regulatory mechanism is the role of itaconate as an endogenous inhibitor of SDH, thereby modulating mitochondrial respiration and preventing excessive inflammatory reactions [75]. Through this mechanism, itaconate mediates profound metabolic reprogramming of the TCA cycle, resulting in dual functional consequences—modulation of macrophage energy metabolism and alteration of inflammatory response capacity [76, 77, 78]. Concurrently, operating within the same metabolic regulatory network, itaconate exerts additional effects by modulating iron homeostasis and disrupting iron–sulfur cluster stability, thereby influencing crucial cellular processes [79]. This metabolic remodeling induced by SDH inhibition further positions itaconate as a critical modulator of macrophage polarization dynamics. Moreover, the regulatory effects of itaconate extend to the oxidative stress axis, with experimental evidence demonstrating that itaconate significantly attenuates intracellular reactive oxygen species (ROS) levels, thereby mitigating oxidative stress‐induced cellular damage and maintaining redox homeostasis [80, 81].

To effectively manage these dynamic changes and maintain cellular homeostasis, macrophages utilize mitophagy to eliminate damaged or dysfunctional mitochondria. This quality control mechanism is crucial for metabolic modulation. In M1 macrophages, mitophagy functions as a negative feedback mechanism to eliminate impaired mitochondria and mitigate excessive or prolonged inflammatory responses [82]. Additionally, by clearing fragmented mitochondria, mitophagy facilitates the metabolic transition toward glycolysis. Conversely, in M2 macrophages, mitophagy is vital for maintaining a healthy mitochondrial population necessary for continuous OXPHOS. This process is orchestrated by pathways involving PTEN‐induced kinase 1 (PINK1) and can be integrated with other metabolic signals. A notable example of such interplay is the enzyme HK2, which directly regulates mitophagy in response to mitochondrial damage [83, 84, 85]. Consequently, the intricate regulation of mitochondrial dynamics, metabolic activity, and quality control mechanisms is fundamental to macrophage polarization. Collectively, these insights establish mitochondrial metabolism as a key therapeutic hub for reprogramming TAMs and reversing tumor‐associated immunosuppression.

## 4 Heterogeneity and Plasticity of TAMs

The polarization state of TAMs is not a fixed endpoint but a highly dynamic and reversible process. This inherent plasticity enables TAMs to continuously adapt their functional phenotypes in response to complex interactions among metabolic cues, epigenetic modifications, and extrinsic signals within the TME. Central to this adaptability are metabolic shifts between different pathways, which provide the bioenergetic foundation necessary for rapid functional reprogramming [86].

### 4.1 Spatial and Temporal Heterogeneity Revealed by Multi‐Omics

**FIGURE 6:**
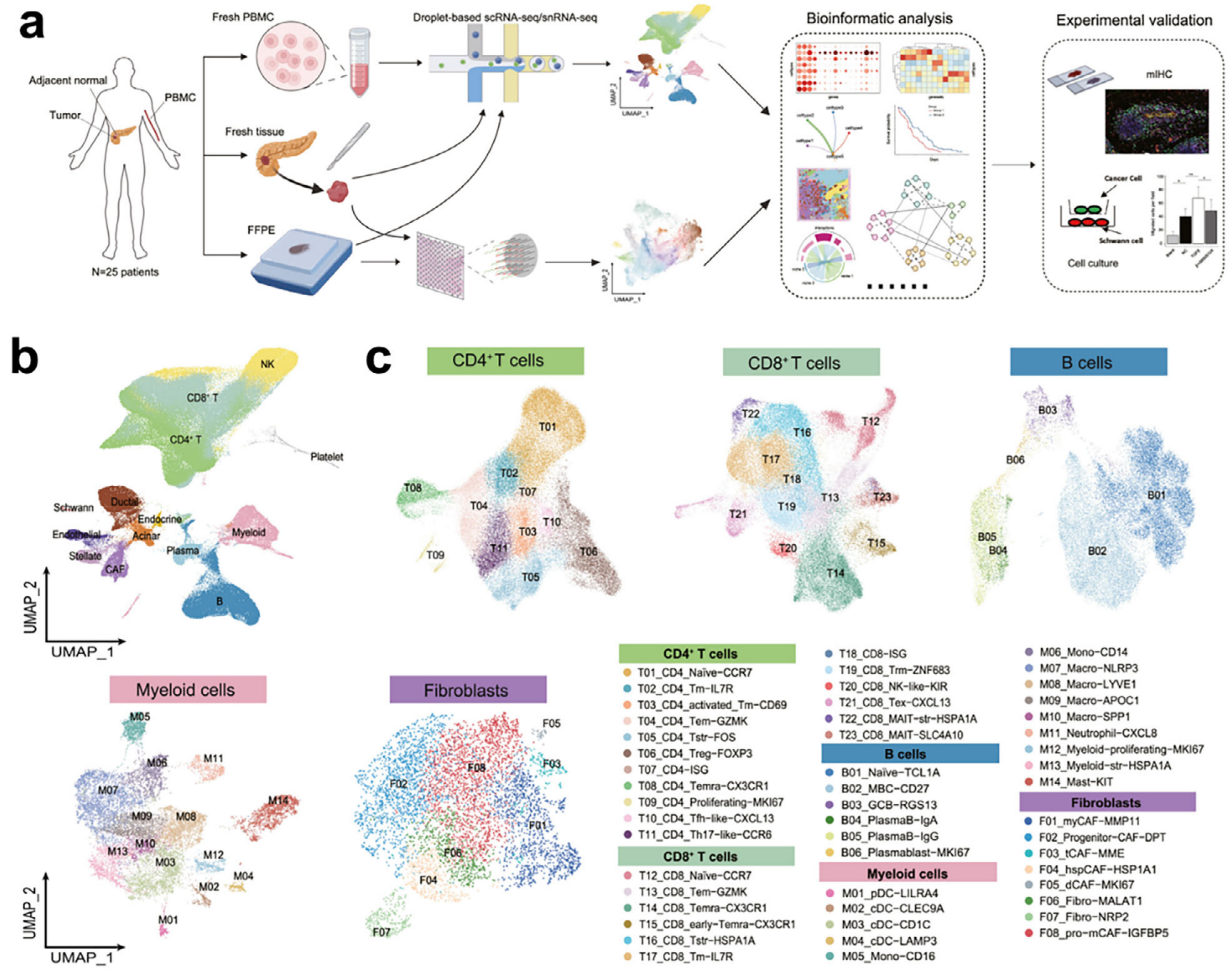
Single‐Cell and Spatial Omics Profiling of Human Pancreatic Ductal Adenocarcinoma (PDAC) tissues. Cell clusters are visualized using Uniform Manifold Approximation and Projection (UMAP) based on scRNA‐seq and snRNA‐seq data. Reproduced with permission [88]. Copyright 2025, Cell Press.

The remarkable heterogeneity of the TME, combined with inter‐tumoral variability, gives rise to a broad spectrum of macrophage phenotypes, rendering the traditional M1/M2 binary classification inadequate for capturing the full functional plasticity of TAMs in vivo [87]. Recent advances in high‐resolution technologies, particularly scRNA‐seq, scATAC‐seq, spatial transcriptomics, proteomics, and metabolomics, have fundamentally reshaped our understanding of TAM biology by revealing that these cells are neither static nor uniformly distributed [88, 89, 90, 91, 92] (Figure 6). Instead, functionally specialized TAM subsets exhibit distinct spatial compartmentalization within tumors, such as perivascular regions, invasive margins, and necrotic areas, closely associated with their local metabolic and cellular niches [87]. Furthermore, these analyses demonstrate that a substantial proportion of TAMs display mixed or intermediate phenotypes. For instance, spatial transcriptomic profiling in glioblastoma has identified subsets such as “interferon‐responsive TAMs” and “lipid‐associated TAMs,” which exhibit pro‐inflammatory and pro‐repair properties, respectively, and show region‐specific distribution [93]. This heterogeneity is clinically relevant, as the presence of mixed‐phenotype TAMs in colorectal cancer positively correlates with patient response rates to immune checkpoint inhibitors [94].

The dynamic evolution of TAMs throughout tumor progression adds another layer of complexity. During early stages, TAMs may retain immune‐surveillance properties, whereas in advanced disease, they predominantly adopt pro‐tumorigenic functions [95]. The convergence of single‐cell transcriptomics with spatial and metabolomic profiling has facilitated a high‐resolution map of TAM heterogeneity, encompassing their ontogeny, functional reprogramming, and spatiotemporal dynamics during tumor progression. For example, single‐cell profiling of hepatocellular carcinoma (HCC) has revealed distinct TAM subsets with divergent origins and functions [96]. TREM2^+^ SPP1^+^ TAMs, predominantly derived from recruited monocytes, are enriched in the peritumoral stroma and invasive margin, where they exhibit immunosuppressive and pro‐fibrotic signatures, including high expression of IL‐10, TGF‐β, and MMP9 [96]. In contrast, folate receptor 2 (FOLR2)^+^ TAMs, originating from embryonic yolk sac progenitors, preferentially localize to perivascular niches within the tumor core and are associated with antigen presentation and T‐cell exclusion. A third subset, lipid‐associated TAMs (LAMs), characterized by high expression of TREM2 and lipid metabolism genes such as FABP4 and LPL, accumulates in necrotic and hypoxic regions and displays a pro‐angiogenic and immunosuppressive phenotype [96].

Integrated spatial and metabolomic analyses further elucidate the metabolic heterogeneity and cellular landscape underlying these observations. For instance, SPP1^+^ TAMs at the invasive front are surrounded by a lactate‐rich metabolic niche originating from glycolytic tumor cells, which reciprocally reinforces their M2‐like polarization via HIF‐1α signaling. Mechanistically, tumor‐cell‐derived lactate is imported into TAMs via MCT1, leading to HIF‐1α/HIF‐2α stabilization, STAT3/AKT activation, and subsequent upregulation of M2‐type genes such as VEGF and ARG1 [97]. Similarly, a metabolism‐driven TAM polarization axis has been identified wherein tumor cells with a “metabolism archetype” upregulate SQLE expression, promoting oxLDL generation; oxLDL then induces TREM2^+^ TAM polarization via the TREM2‐SYK‐CEBPα axis, thereby promoting cancer cell invasion and CD8^+^ T cell dysfunction [98]. These integrated approaches unveil a complex, spatially organized interaction network among tumor cells, hepatocytes, and distinct TAM subsets, offering a novel spatial biology perspective on mechanisms underlying liver cancer invasion.

Collectively, these dynamic single‐cell and spatial multi‐omics platforms constitute powerful tools for investigating immune‐tumor metabolic crosstalk at single‐cell resolution. Their application has not only deepened our understanding of TAM heterogeneity and functional diversity but also identified novel therapeutic targets‐such as TREM2, SPP1, FOLR2, and components of lipid metabolic pathways (FABP4, LPL, SQLE, GPX4)‐and provided a conceptual framework for developing immunotherapy strategies centered on metabolic reprogramming [99].

### 4.2 Epigenetic Modifications Confer TAM Plasticity

Epigenetic mechanisms, such as DNA methylation and histone modifications, establish a poised transcriptional landscape that enables rapid phenotype switching [100]. A key example is the IL‑4‑induced expression of M2‑associated genes, such as *ARG1* and *MRC1* (encoding CD206), which is promoted by DNA demethylation at their promoter regions‐a process regulated by DNA methyltransferases (DNMTs) [101]. In pancreatic ductal adenocarcinoma (PDAC) tumors, direct cell–cell interactions induce DNA methylation and transcriptional downregulation of a panel of genes involved in glucose metabolism and OXPHOS in M1‐like macrophages, but not in M2‐like macrophages [102]. However, blocking DNMT activity with decitabine can effectively restore immunosurveillance capacity [40].

Histone modifications, including methylation, acetylation, and lactylation, provide an additional synergistic layer of epigenetic regulation over macrophage polarization. M1 polarization is associated with activating marks such as H3K4me3 at the promoters of pro‐inflammatory genes (e.g., *NOS2*), whereas M2 polarization is reinforced by repressive marks like H3K27me3, which silence inflammatory pathways to promote an anti‑inflammatory, pro‑tumorigenic phenotype [103]. This malleable epigenetic landscape enables various signals within the TME to actively repolarize TAMs. For example, mesenchymal stem cells (MSCs) can secrete IL‐4 and IL‐13, which induce TAMs toward an M2 phenotype via the IL‐4Rα/JAK1/STAT6 pathway [104]. Notably, the JAK/STAT pathway is a highly conserved signaling cascade involved not only in immunoregulation but also intimately associated with the pathogenesis of various human diseases, as it regulates multiple cellular processes [105, 106]. Beyond its role in TAM polarization, this pathway has been extensively targeted in clinical oncology. Clinical studies have demonstrated that the STAT inhibitor OPB‐111077 and decitabine can inhibit cancer cell growth by targeting enzymes essential for cell proliferation. Chemotherapy drugs such as venetoclax suppress cancer cell growth through different mechanisms, including inducing cell death, preventing cell division, or inhibiting metastasis. Combining the STAT inhibitor OPB‐111077, decitabine, and venetoclax may be more effective in treating acute myeloid leukemia than using decitabine alone (NCT03063944) [107]. These clinical findings underscore the broad therapeutic potential of targeting the epigenetic modifications, which plays a crucial role in the onset and progression of many disorders [108].

### 4.3 TME‐Derived Signals Driving TAM Plasticity

Metabolic byproducts within the TME also act as potent signaling molecules. In hypoxic tumor regions, the accumulation of adenosine triggers the expression of A2A receptor (A2AR) on macrophages. This signaling can override prior Toll‐like receptor (TLR) activation, converting pro‐inflammatory M1‐like macrophages into an immunosuppressive M2‐like phenotype that inhibits CD8^+^ T cell infiltration [109]. Activation of the adenosine A2AR signaling pathway promotes immunosuppression, epithelial‐mesenchymal transition in tumors, and resistance to apoptosis, thereby facilitating tumor progression [110, 111] Currently, several dual adenosine A2AR/A2BR inhibitors, such as AB928 and YZJ‐5053, are being investigated in multiple clinical trials. Studies have demonstrated that combining AB928 with agents such as PD‐1 monoclonal antibodies, TIGIT monoclonal antibodies, or CD73 inhibitors may be effective in treating various cancers, including prostate, colorectal, non‐small cell lung, and pancreatic cancer [112]. A randomized, open‐label Phase 1b/2 study is ongoing to evaluate the antitumor activity and safety of Etrumadenant(AB928) combination therapy in patients with metastatic colorectal cancer (NCT04660812). Additionally, another study is assessing the safety, tolerability, pharmacokinetics, and efficacy of YZJ‐5053 tablets in patients with advanced solid tumors (NCT06209385). Therefore, the development of novel A2AR inhibitors is critically important for advancing cancer immunotherapy.

In summary, the plasticity of TAMs is a multifaceted process governed by epigenetic modification and signaling control. While essential for physiological processes like inflammation resolution and tissue repair, this dynamism is co‑opted within tumors to foster immune evasion. Consequently, a deeper understanding of these regulatory hubs establishes a rational foundation for developing therapies that reprogram TAMs to reinstate anti‐tumor immunity.

## 5 Therapeutic Strategies Targeting TAM Metabolism

**FIGURE 7:**
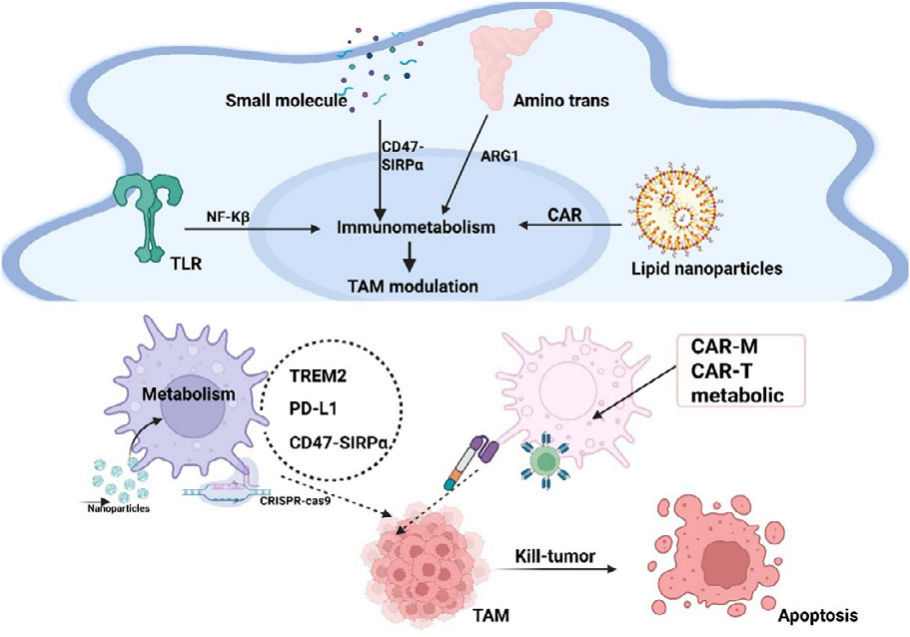
Immunotherapy strategies targeting TAM metabolism. Created in https://BioRender.com.

The functional state of TAMs is closely linked to metabolic reprogramming within the TME. This connection is evidenced by the distinct metabolic pathways that TAMs employ in different polarization states. For instance, a metabolic shift from glycolysis to fatty acid oxidation not only determines the functional phenotype of TAMs but also influences the homeostasis of the entire TME [113]. Altered metabolic states often drive TAMs to secrete a variety of cytokines and angiogenic factors, thereby directly or indirectly promoting tumor growth, invasion, and immune evasion [114, 115]. Consequently, targeting and regulating TAM metabolism has emerged as a critical strategy to modulate their function and remodel the tumor immune microenvironment, representing a promising avenue in cancer immunotherapy [116] (Figure 7).

### 5.1 Targeting Key Metabolic Pathways and Molecular Nodes

#### 5.1.1 Modulators of Fatty Acid Metabolism

Lipid metabolism encompasses both the catabolism and anabolism of lipids [117]. It is crucial for the polarization and function of TAMs, presenting several promising avenues for therapeutic intervention [118]. In HCC, a distinct population of lipid‐droplet‐laden macrophages (LLMs) accumulates in tumor tissues, exhibiting immunosuppressive phenotypes characterized by high expression of TREM2, PD‐L1, CD206, and CD163, along with impaired CD8^+^ T‐cell activity. Targeting the triglyceride synthesis pathway by inhibiting diacylglycerol acyltransferases DGAT1 and DGAT2 significantly reduces Treg recruitment and delays tumor growth in preclinical models, establishing lipid synthesis as a viable therapeutic target [119].

Beyond triglyceride metabolism, cholesterol homeostasis represents another critical regulatory node, as demonstrated in ovarian cancer models where cancer cells promote membrane cholesterol efflux from macrophages. This process depletes lipid rafts, enhances IL‐4–mediated reprogramming, and inhibits IFN‐γ–induced gene expression. Genetic deletion of ATP‐binding cassette (ABC) transporters, which mediate cholesterol efflux, effectively reverses the tumor‐promoting functions of TAMs and reduces tumor progression, revealing an unexpected therapeutic opportunity [56]. Notably, the regulation of lipid metabolism serves as a core mechanism in cellular immune responses, ultimately exerting anti‐tumor effects through immune activation.

Recent advances in targeted delivery approaches have shown significant promise in modulating lipid metabolism within TAM. Nanoparticles incorporating both viral RNA analogs and the FAO regulator cryptotanshinone (CTS) have demonstrated the ability to reprogram tumor‐infiltrating macrophages from an immunosuppressive M2‐like phenotype to a pro‐inflammatory M1 state [120]. This metabolic reprogramming not only enhances the secretion of pro‐inflammatory cytokines but also promotes the recruitment of CD8^+^ T cells to the tumor microenvironment. Moreover, the fatty acid oxidation pathway offers additional intervention points through transcriptional regulators like Dual Specificity Phosphatase 5 (DUSP5), which functions downstream of TLR2‐MAPK signaling to promote FAO in macrophages. Silencing DUSP5 increases free fatty acid content and triglyceride levels while repressing expression of FAO associated transcripts and enzymes including CPT1A and PPAR‐α [46]. Similarly, direct pharmacologic inhibition of FAO demonstrates anti‐inflammatory effects, suggesting that strategic modulation of lipid metabolism pathways may offer therapeutic avenues for reshaping the immunosuppressive tumor microenvironment, thereby enhancing antitumor efficacy.

#### 5.1.2 Modulators of Amino Acid Metabolism

Amino acid availability profoundly influences the function of TAMs, making key metabolic enzymes in these pathways attractive therapeutic targets. A prominent example is the targeting of glutaminase, a central enzyme in glutamine metabolism that critically regulates macrophage polarization within the TME. In preclinical models, such as triple‑negative breast cancer, glutaminase inhibitor CB‑839 exerts antiproliferative effects by significantly reducing glutamine consumption, glutamate production, and cellular oxygen utilization [121]. Beyond its direct impact on tumor cells, glutamine metabolism is intricately linked to macrophage function [122]. Glutamine serves as a precursor for glutathione synthesis and modulates the inflammatory status of macrophages, suggesting that glutaminase inhibition may simultaneously disrupt tumor metabolism and reprogram TAM phenotypes.

Moreover, Arg‐1 is highly expressed in TAMs and MDSCs and plays a central role in establishing immunosuppression. In renal cell carcinoma, MDSCs expressing elevated levels of Arg‑1 deplete extracellular L‑arginine, thereby inducing T‑cell anergy, impairing T‐cell proliferation and cytokine production, and downregulating the T‑cell receptor CD3ζ chain [123]. This immunosuppressive mechanism is further corroborated in pancreatic ductal adenocarcinoma, where high Arg‑1 expression correlates with poorer patient survival. The therapeutic potential of targeting this pathway was demonstrated by the validation of Arg‐1 genetic deletion in preclinical models. Genetic deletion of Arg‑1 in myeloid cells reduced tumor progression and enhanced CD8^+^ T‑cell infiltration in pancreatic cancer. More importantly, pharmacological inhibition of arginase using CB‐1158, particularly in combination with anti‐PD1 immune checkpoint blockade, significantly reduced tumor burden in orthotopic pancreatic cancer models [64].

In addition to direct pharmacological inhibition strategies, an alternative approach involves harnessing the immune system to target Arg1. Arg1‑targeting immune modulatory vaccines (IMVs) can control tumor growth by altering the M1/M2 macrophage ratio [124]. Mechanistically, Arg‐1‐specific CD4^+^ T cells directly recognize and eliminate Arg‑1‑expressing TAMs. Through the secretion of IL‑2 and IFN‑γ, these T cells reprogram targeted TAMs toward a pro‑inflammatory M1‑like state, as evidenced by a significant reduction in CD206^+^ macrophages following Arg‑1 IMV treatment [125].

While targeting amino acid‐metabolizing enzymes offers promising therapeutic opportunities, careful consideration must be given to potential impacts on immune cell function. For instance, glutaminase inhibition with CB‐839 has been shown to suppress the clonal expansion and activation of CD8^+^ T cells, potentially weakening anti‐tumor immunity [69]. These findings highlight the complex interplay among amino acid metabolism, macrophage polarization, and T‑cell function within the tumor microenvironment. Therefore, the design of metabolic interventions must aim to selectively reprogram TAMs while preserving or even enhancing effector T‑cell responses.

### 5.2 Immunomodulatory Agents

#### 5.2.1 TLR Agonists

TLR agonists reprogram TAMs toward an anti‐tumor phenotype by activating innate immune signaling pathways and inducing metabolic rewiring. Upon stimulation with agonists such as LPS, macrophages undergo a profound metabolic shift from OXPHOS to aerobic glycolysis. This reprogramming is characterized by increased lactate production, enhanced PPP activity, and decreased TCA cycle function, collectively establishing the bioenergetic foundation for robust inflammatory responses. The TLR induced metabolic shift is orchestrated by the transcription factor HIF‑1α, which plays a critical role by binding to hypoxia‐response elements in the promoters of genes encoding glucose transporters and glycolytic enzymes [126], thereby facilitating this metabolic switch within the heterogeneous tumor microenvironment. Notably, TLR‑induced glycolytic reprogramming is essential for effective macrophage activation. While treatment with TLR agonists alone induces robust inflammatory responses, the addition of 2‑deoxy‑D‑glucose (2‑DG) significantly attenuates this effect [31].

The metabolic shift orchestrated by TLR signaling can even induce critical alterations in the processing of TCA cycle intermediates, notably citrate and succinate. TLR activation upregulates the mitochondrial citrate carrier (Slc25a1) via NF‑κB signaling, facilitating citrate export from mitochondria and its subsequent accumulation in the cytosol [127]. This redistributed citrate serves as a vital precursor for fatty acid synthesis and protein acetylation, processes essential for macrophage inflammatory function. Concurrently, TLR stimulation elevates succinate levels, which stabilizes HIF‑1α, thereby reinforcing glycolytic metabolism and promoting inflammatory gene expression [31].

Beyond these canonical changes, recent studies reveal a complementary role for mitochondrial FAO in fine‑tuning TLR responses through epigenetic mechanisms. The enzyme acetyl‑CoA acetyltransferase 1 (ACAT1), which mediates FAO, generates acetyl‑CoA required for histone acetylation at promoters of interferon‑stimulated genes. Depletion of ACAT1 diminishes histone acetylation and impairs type I interferon signaling, highlighting a crucial metabolic‑epigenetic axis in macrophage activation [55]. This axis represents a promising therapeutic target, particularly given the enhanced efficacy observed when combining TLR agonists with immune checkpoint blockade in preclinical models [128].

Another example of TLR‑mediated metabolic reprogramming involves CpG oligodeoxynucleotides (CpG‑ODNs), synthetic TLR9 agonists [129] (Table 1). Clinically, CpG‑ODNs have shown promise in maintaining therapeutic efficacy when combined with radiotherapy, chemotherapy, or immune checkpoint inhibitors, as evidenced by several trials (e.g., NCT00043407, NCT00070642) [130, 131]. Further refinement of TLR‑targeted strategies is possible through chemical conjugation of TLR agonists to other immunomodulatory molecules, which can optimize their pharmacokinetics, toxicity profile, and overall efficacy [132]. Such precision engineering approaches hold significant potential for advancing TLR‑mediated metabolic reprogramming of TAMs as a viable cancer immunotherapy.

**TABLE 1:** Summary of cancer therapies with metabolic implications.

Therapeutic Category	Drug/Agent (Examples)	Target / Mechanism of Action	Key Immunomodulatory Effects	Clinical trials	Conditions	Sponsor	Gov identifier
Signaling Pathway Inhibitor Combined with Immune Checkpoint Inhibitor	Metformin with Anti‐PD‐1/PD‐L1 Antibody	Activation of AMPK; T cell exhaustion	Promotes differentiation of MDSCs into dendritic cells (DCs) [139, 140].	Phase 2	Breast Cancer	Tanta University	NCT05053841
TLR agonists	CpG 7909	Inhibited TCA cycle, upregulated glycolysis	Reprogrammed M2 to M1; Targeting metabolic pathway.	Phase 1/2 Phase 2	Renal cell carcinoma Melanoma	Pfizer	NCT00043407; NCT00070642
Signaling Pathway Inhibitor	Idelalisib	Blocks the PI3K/Akt pathway	Reduces M2‐like polarization of TAMs [141]; Enhances CD8^+^ and CD4^+^ T cell activity.	Phase 2	Refractory Hodgkin Lymphoma	Gilead Sciences	NCT01393106
Signaling Pathway Inhibitor	Ruxolitinib	Inhibits JAK/STAT phosphorylation	Blocks IL‐6‐mediated tumor angiogenesis; Enhances anti‐tumor activity in various cancers [106, 142].	Phase 2	Premalignant Breast Disease	Julie Nangia	NCT02928978
Epigenetic Regulator HDAC Inhibitors	Romidepsin	Epigenetic regulation of gene expression	Promotes M1‐like polarization; Enhances macrophage phagocytosis [143].	Phase 2	Small cell lung cancer	National Cancer Institute	NCT00086827
CD47‐SIRPα Blockade	CC‐90002	Anti‐CD47 monoclonal antibody	Targeting the CD47‐SIRPα pathway promotes macrophage clearance of tumor cells [134, 135].	Phase 1	Refractory solid and hematologic cancers.	Celgene	NCT02367196
Signaling Pathway Inhibitor	BBI608	Inhibits STAT3 signaling	Directly kills liver cancer stem cells [144]; Reverses TME immunosuppression.	Phase 1	Advanced malignancie; Glioblastoma	Sumitomo Pharma America, Inc.	NCT01775423; NCT02315534

#### 5.2.2 CD47‐SIRPα Blockade

A key strategy to harness macrophage anti‐tumor function is to disrupt the inhibitory signals that tumor cells exploit to evade phagocytosis. The CD47‐SIRPα axis exemplifies a major “don't eat me” immune checkpoint, in which CD47, which is often overexpressed on tumor cells, binds to SIRPα on myeloid cells, such as macrophages, to suppress phagocytic activity [133]. High CD47 expression correlates with poor prognosis, making this axis a compelling therapeutic target [133]. For example, the humanized anti‑CD47 antibody CC‑90002 binds CD47 with high affinity, effectively blocks the CD47‐SIRPα interaction, and exhibits a favorable safety profile without inducing hemagglutination [134]. Preclinical studies demonstrate that CC‐90002 promotes concentration‑dependent phagocytosis in multiple hematological cancer cell lines, including acute lymphoblastic leukemia and acute myeloid leukemia, as well as in advanced solid and hematologic cancers (NCT02367196) [135, 136]. In vivo studies have further confirmed its dose‐dependent antitumor activity and its ability to prolong survival in disseminated AML models through macrophage recruitment and activation [134].

Furthermore, synergistic potential arises when CD47‐SIRPα blockade is combined with agents that provide a pro‐phagocytic “eat me” signal. For example, combining this blockade with STING agonists or incorporating it into chimeric antigen receptor (CAR) constructs has been shown to significantly enhance tumor cell phagocytosis and induce systemic anti‐tumor immunity in models where monotherapy is insufficient [133]. Beyond checkpoint blockade, numerous other antibodies and small‑molecule compounds are being developed to reprogram M2‑like macrophages toward an M1‑like phenotype. These agents employ diverse strategies‐such as direct phenotypic reprogramming, disruption of energy metabolism, or functional impairment of M2‑like TAMs‐to effectively reverse immunosuppression and activate a broad anti‐tumor immune response.

Despite compelling preclinical rationale, the clinical translation of CD47‐targeting therapies has faced significant challenges and several high‐profile failures. The development of multiple agents, including ZL‐1201, has been terminated or deprioritized due to safety concerns and lack of efficacy [137]. The most significant obstacle arose from the clinical trials of Magrolimab. Its phase III ENHANCE study, which evaluated magrolimab combined with azacitidine in high‐risk myelodysplastic syndromes (MDS), was halted due to lack of clinical benefit and an increased risk of death in the treatment arm [138]. Similar futility and increased mortality led to the termination of its development in acute myeloid leukemia (AML). These repeated failures have sparked intense debate regarding the druggability of CD47 and the viability of this therapeutic approach, particularly in hematological malignancies. However, these clinical setbacks do not invalidate the CD47‐SIRPα axis as a target. Rather, they underscore that merely blocking the “don't eat me” signal is insufficient, especially in heavily pretreated or high‐risk patients. To overcome the limitations observed in the clinic, CD47‐SIRPα blockade likely needs to be integrated with complementary therapeutic mechanisms. Promising approaches include reprogramming TAMs to an M1‐like state via STING agonists, or synergizing with CAR‑based therapies to achieve robust anti‐tumor activity.

### 5.3 Advanced Platforms for TAM Reprogramming

#### 5.3.1 Nanotechnology and Biomimetic Delivery Systems

Advanced delivery platforms, including biomimetic nanosystems and other nanoparticle‐based technologies, have emerged as versatile tools for the targeted delivery of metabolic modulators, genetic material, and therapeutic agents specifically to TAMs. These platforms offer a promising approach to enhance therapeutic efficacy while minimizing off‐target effects [145]. Building on this capability, recent advances in nanoparticle engineering have enabled the design of sophisticated systems that precisely reprogram TAMs by targeting their metabolic pathways and polarization states, thereby actively reshaping the tumor immune microenvironment [146, 147, 148]. A promising strategy involves engineered M1 macrophage‐derived exosomes surface‐modified with IL4RPep‐1, an IL‐4 receptor‐binding peptide, to specifically target TAMs expressing high levels of the IL‐4 receptor. These exosomes efficiently migrate to tumor sites and reprogram TAMs into M1‐like macrophages through the co‐delivery of NF‐κB p50 siRNA and miR‐511‐3p, significantly inhibiting tumor growth and enhancing antitumor immunity [149].

**FIGURE 8:**
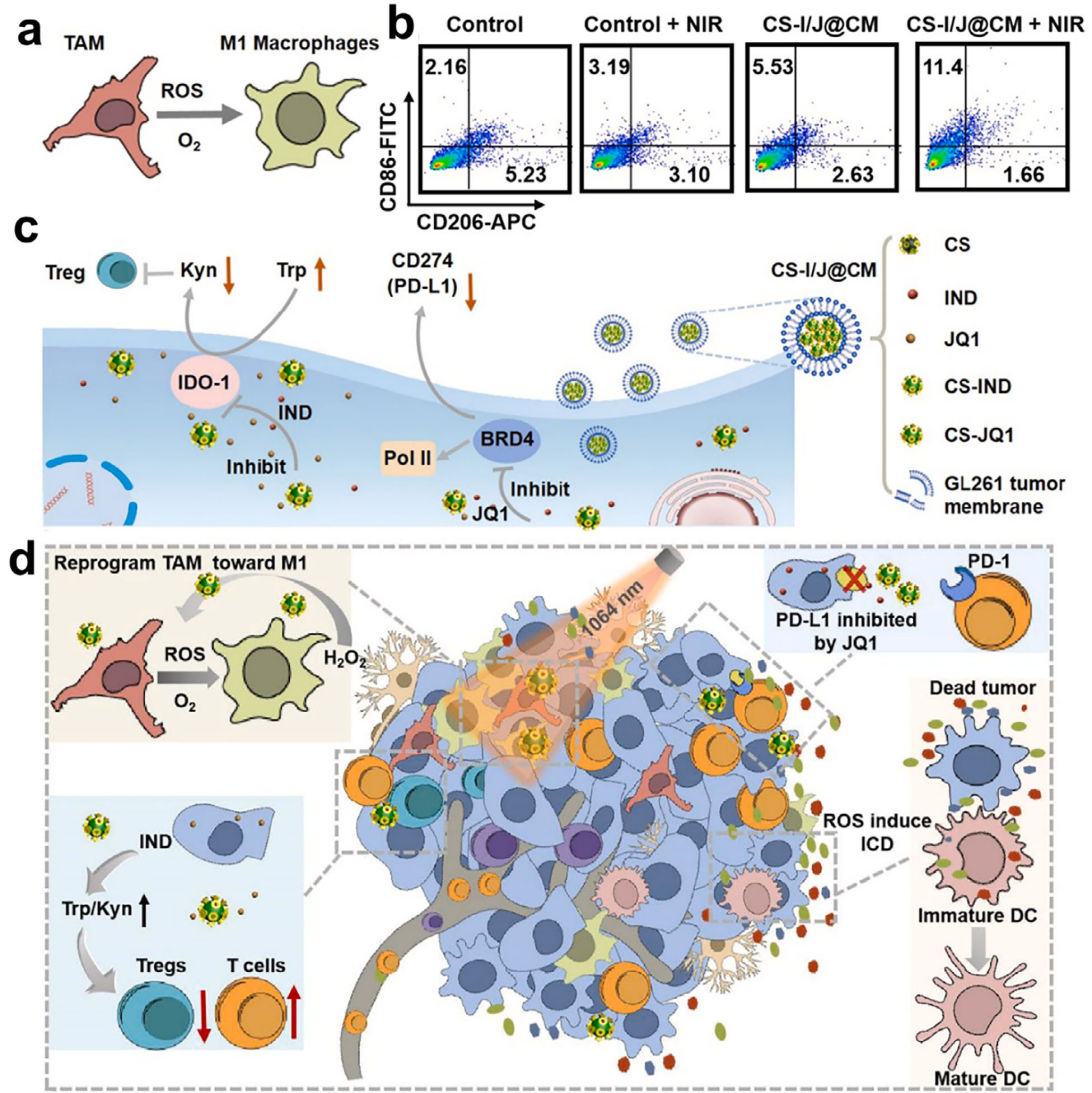
The immunotherapeutic mechanism of CS‐I/J@CM NPs in the treatment of glioblastoma by reshaping the tumor immunosuppressive microenvironment. Reproduced with permission [150]. Copyright 2022, Elsevier.

Another approach focuses on altering the physicochemical properties of the TME. The hypoxic microenvironments that drive M2 polarization can be directly reversed using biomimetic nanoparticles (CS‐I/J@CM NPs) [150]. These nanoparticles catalyze Fenton‐like reactions to generate both oxygen and reactive oxygen species (ROS) under near‐infrared irradiation. This dual action not only alleviates tumor hypoxia but also effectively repolarizes TAMs to an M1 phenotype. Notably, these nanoparticles induce a remarkable four‐fold increase in tumor‐infiltrating effector memory T cells (Figure 8).

**FIGURE 9:**
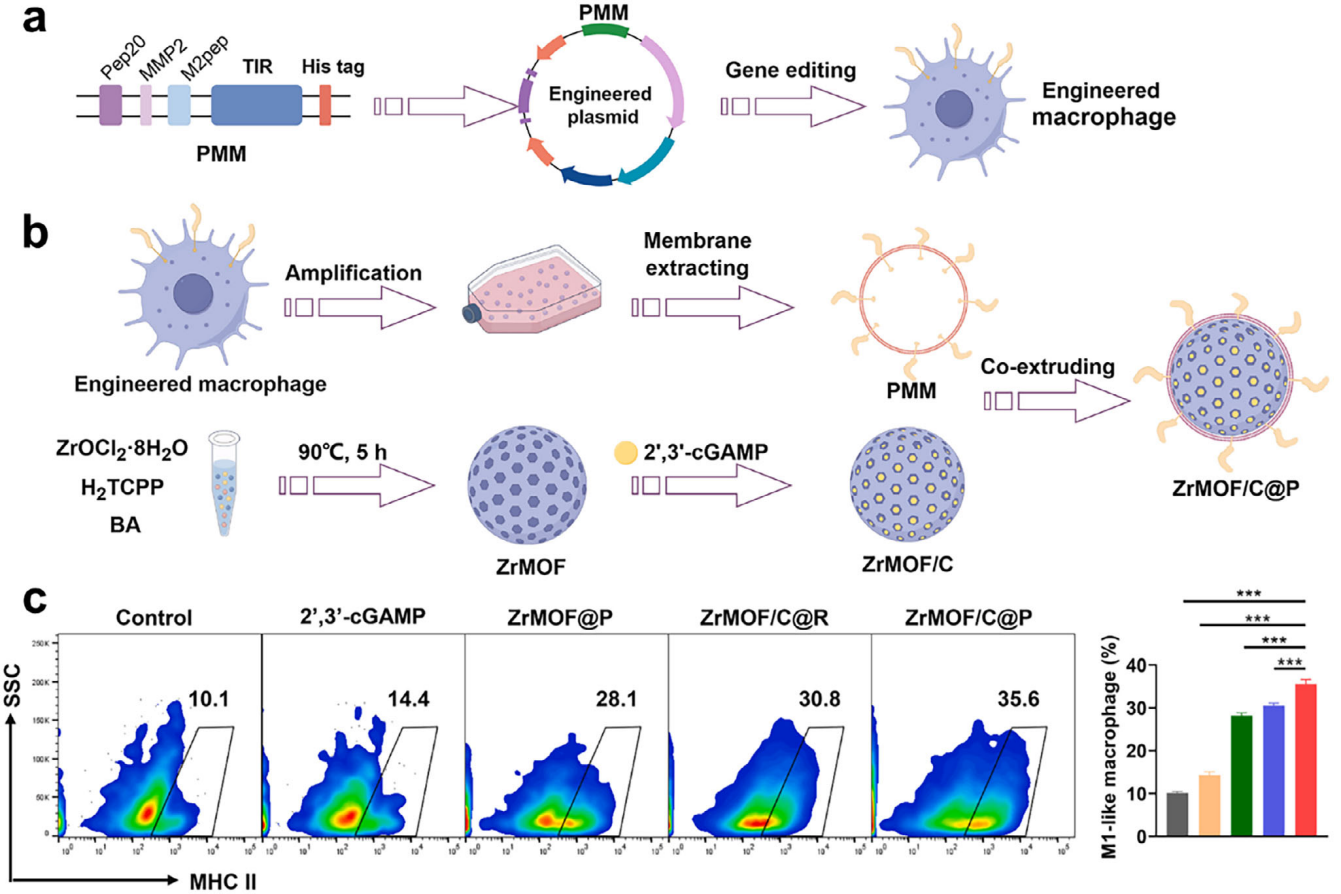
Synthesis of ZrMOF/C@P intelligent nanoparticles and their targeting to TAMs. Reproduced with permission [133]. Copyright 2025 Elsevier.

The most advanced platform integrates multiple treatment modalities into a single “smart” system [133, 151]. A notable example is a zirconium‐based metal–organic framework (ZrMOF) engineered for multi‐stage targeting and action (Figure 9). Coated with a macrophage membrane decorated with M2‐targeting peptides, this nanoparticle is selectively taken up by M2‐like TAMs. Once inside the TME, it releases a peptide that blocks the CD47‐SIRPα interaction, thereby restoring macrophage phagocytic function. Simultaneously, a loaded STING agonist is released, activating the STING pathway within the TAMs. This combination of enhanced phagocytosis and metabolic reprogramming induces a potent anti‐tumor M1 phenotype, as evidenced by an approximately 25‐fold increase in IFN‐β expression [133].

Innovative biomaterials hold great promise for reprogramming. Various platforms are exploring iron oxide nanoparticles, calcium‐based nanoplatforms, and even the integration of traditional Chinese medicine with nanomaterials to synergistically reverse TME immunosuppression [152, 153, 154]. Collectively, these advanced nanoparticle strategies represent a paradigm shift in cancer immunotherapy. Their unprecedented precision and multifunctionality enable the strategic manipulation of macrophage metabolism and function, effectively converting immunosuppressive cells into potent anti‑tumor effectors and reshaping the overall immune landscape of the TME.

#### 5.3.2 Gene Editing (CRISPR‐Cas9) and CAR‐Macrophages

CRISPR‐Cas9‐mediated precision base editing represents a revolutionary strategy for the precise modulation of metabolic pathways that regulate TAM function, both in vivo and in vitro [155]. This technology enables the targeted knockout of pro‑tumorigenic genes or the insertion of anti‑tumor effectors directly within TAMs. A prominent non‑viral delivery platform is CRISPR‑Gold, which utilizes gold nanoparticles to efficiently deliver Cas9 ribonucleoproteins (RNPs) and donor DNA into macrophages, facilitating homology‑directed repair (HDR) [156]. This strategy achieves high editing efficiency in primary cells with minimal off‑target effects. Moreover, systemic cytokine profiling indicates that CRISPR‑Gold does not induce acute upregulation of inflammatory cytokines in plasma, supporting its potential for safe and repeated administration in therapies aimed at reprogramming macrophage metabolism.

**FIGURE 10:**
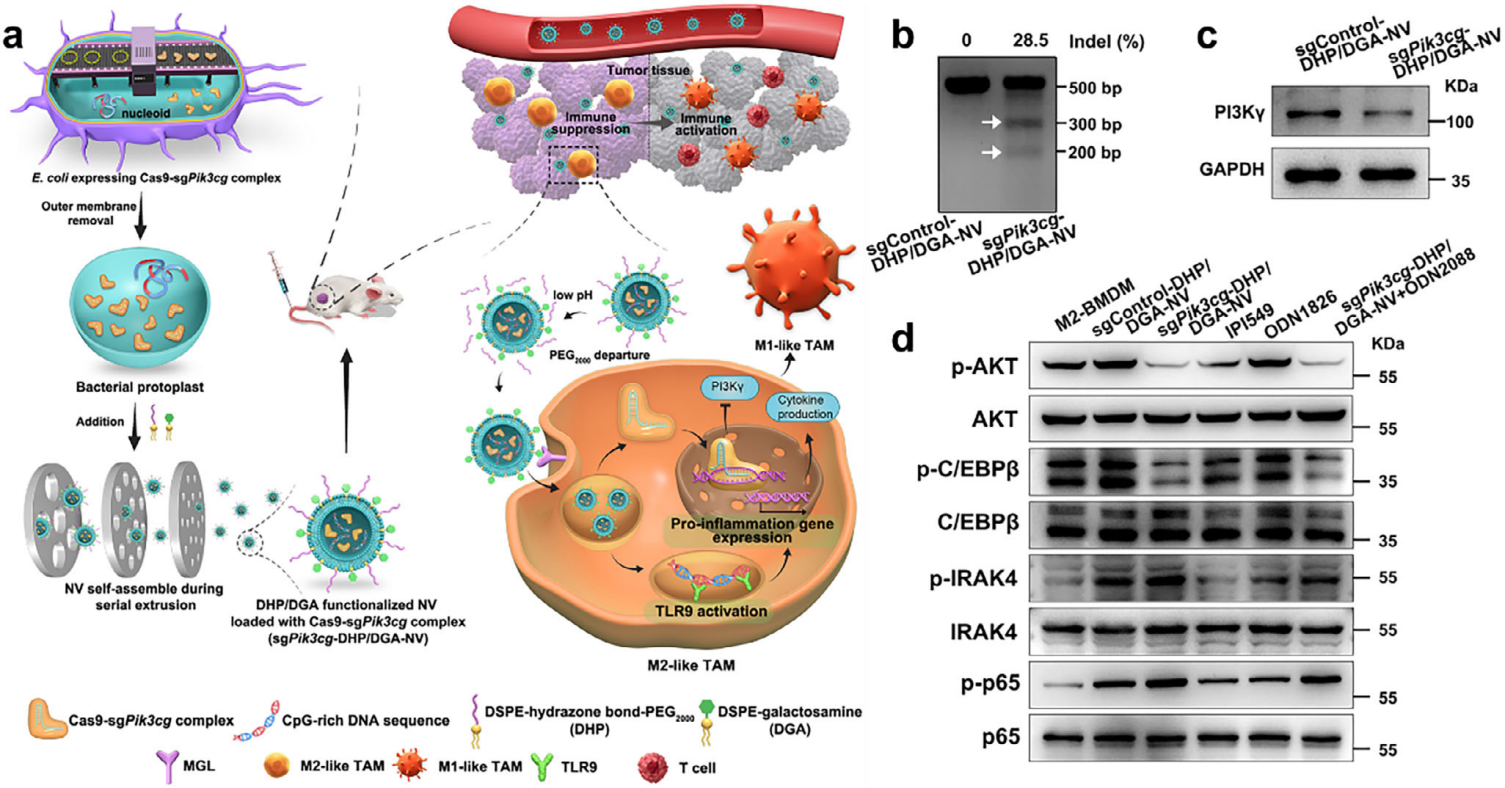
CRISPR‐Cas9‐mediated reprogramming of TAMs. Reproduced with permission [10]. Copyright 2024, Nature.

Bacterial vesicle‐based delivery systems represent another highly promising vehicle for CRISPR delivery due to their ability to target macrophages (Figure 10). Pre‐assembled Cas9/sgRNA RNP complexes can be delivered effectively using this method, as demonstrated by significantly higher editing efficiency and target specificity compared to plasmid‐based approaches [10]. These advanced delivery systems open the door to sophisticated in situ reprogramming strategies, such as knocking out key M2‐polarization genes (e.g., *STAT6*), targeting metabolic regulators (e.g., *HIF1A*), or engineering TAMs to overexpress pro‐inflammatory factors like IFN‐γ.

Engineering macrophages to function as a “living drug” constitutes an effective new cancer therapy with significant therapeutic potential [157]. In this strategy, macrophages are transduced with a viral vector to express a chimeric antigen receptor (CAR) targeting a tumor‐specific antigen, generating CAR‐macrophages (CAR‐Ms). Notably, engagement with antigen‐expressing cancer cells induces metabolic reprogramming in CAR‐M cells, characterized by increased glycolytic activity and reduced oxidative phosphorylation, thereby enhancing their anti‐tumor efficacy [158].

Collectively, these cutting‐edge gene and cell engineering technologies are being integrated to overcome the inherent limitations of conventional therapies. By directly reprogramming the genetic or cellular functions of macrophages, they offer the potential to transform pro‐tumorigenic TAM populations into potent and durable anti‐tumor effectors, thereby opening a new and powerful avenue for cancer treatment.

#### 5.3.3 Targeted Protein Degradation (PROTACs)

Targeted protein degradation (TPD) has emerged as a novel strategy in molecularly targeted therapy, utilizing cellular degradation pathways such as the ubiquitin‐proteasome system (UPS) and autophagy to selectively eliminate target proteins. Among TPD approaches, proteolysis‐targeting chimeras (PROTACs) are the earliest and most extensively studied, enabling the development of novel degraders including AUTACs, LYTACs, and AbTACs. Unlike conventional small‐molecule inhibitors, PROTACs induce target degradation through an event‐driven mechanism. This distinctive feature makes them a promising alternative for overcoming resistance in molecularly targeted cancer therapies [92].

PROTACs are heterobifunctional molecules that mediate targeted protein degradation via the UPS [159]. By hijacking this pathway, PROTACs can exploit the ubiquitin‐proteasome system to selectively degrade target proteins and induce metabolic rewiring in macrophages [160]. For example, the degradation of HK2 facilitated by PROTAC significantly inhibits glycolytic activity as well as mitochondrial respiration, consequently diminishing oxygen consumption in the TME during photodynamic therapy [161].

## 6 Challenges and Further Directions

Despite promising advances in targeting TAM metabolism, significant challenges remain in optimizing these approaches for clinical efficacy. The inherent heterogeneity of TAM populations presents a fundamental obstacle, as macrophage functional phenotypes within tumor tissues often vary between individuals and even among different regions of the same tumor [4]. This heterogeneity also extends to metabolic programming, with distinct TAM subpopulations exhibiting varied metabolic signatures that dynamically respond to localized microenvironmental cues. Therefore, understanding these complex metabolic processes requires integrating multi‐omics approaches that simultaneously assess gene expression, chromatin accessibility, and metabolite dynamics to comprehensively map how metabolic reprogramming influences epigenetic and transcriptional regulation in macrophages [55].

Moreover, TME is not a process in which the tumor unidirectionally determines the phenotype of macrophages. On the contrary, there is a metabolic exchange between tumor cells and TAMs [162, 163]. The hostile tumor microenvironment, characterized by hypoxia and acidosis, profoundly exacerbates this inherent complexity and complicates therapeutic efforts by promoting M2‑like polarization while suppressing anti‑tumor immune effector function [4]. Consequently, future therapeutic approaches must transcend a narrow focus on macrophage‐intrinsic metabolic pathways. Integrating these multifaceted insights into macrophage metabolic plasticity with precision immunomodulatory strategies represents a promising frontier for enhancing anti‐tumor immunity and improving clinical outcomes across various cancer types.

## Author Contributions

Z.Y.L., L.Y.Z., X.S., and L.Y.S. conceived the topic, drew up the outline, wrote and revised the original draft. All authors reviewed and edited the draft. X.S., D.W., and L.Y.S. provided research funding for the project. Z.Y.L., L.Y.Z., and X.S. produced the figures and tables of the manuscript. All authors read and approved the final manuscript.

## Conflicts of Interest

The authors declare no conflicts of interest.
